# 2-(4-Carb­oxy­piperidinium-1-yl)pyridine-3-carboxyl­ate

**DOI:** 10.1107/S1600536812005752

**Published:** 2012-02-29

**Authors:** Ping Fan, Chunhua Ge, Mingjun Sun, Weiwei Li, Runshan Shang

**Affiliations:** a College of Chemistry, Liaoning University, Shenyang 110036, People’s Republic of China

## Abstract

The title compound, C_12_H_14_N_2_O_4_, crystallizes as a zwitterion. A negative charge is delocalized in the deprotonated carboxyl group attached to the pyridine ring. The piperidine N atom accepts a proton and the ring is transformed into a piperidinium cation. There is an intra­molecular N—H⋯O hydrogen bond between the protonated NH and a carboxyl­ate O atom. In the crystal, an O—H⋯O hydrogen bond between the carboxyl group and the carboxyl­ate O atom of another mol­ecule generates a helix along the *b* axis.

## Related literature

For the synthesis, see: Shreder *et al.* (2009 ▶); Léost *et al.* (1997 ▶); Bonnet *et al.* (2002 ▶).
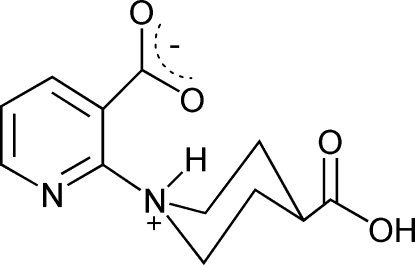


## Experimental

### 

#### Crystal data

C_12_H_14_N_2_O_4_
*M* *_r_* = 250.25
Monoclinic, 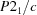

*a* = 7.1094 (14) Å
*b* = 18.667 (4) Å
*c* = 8.6603 (17) Å
β = 93.57 (3)°
*V* = 1147.1 (4) Å^3^
*Z* = 4
Mo *K*α radiation
μ = 0.11 mm^−1^
*T* = 293 K
0.20 × 0.20 × 0.20 mm

#### Data collection

Bruker SMART CCD area-detector diffractometer
Absorption correction: multi-scan (*SADABS*; Bruker, 2001 ▶) *T*_min_ = 0.964, *T*_max_ = 0.983
6010 measured reflections
2237 independent reflections
1841 reflections with *I* > 2σ(*I*)
*R*_int_ = 0.022

#### Refinement

*R*[*F* ^2^ > 2σ(*F* ^2^)] = 0.042
*wR*(*F* ^2^) = 0.118
*S* = 1.09
2237 reflections
168 parameters
H atoms treated by a mixture of independent and constrained refinement
Δρ_max_ = 0.22 e Å^−3^
Δρ_min_ = −0.34 e Å^−3^

### 

Data collection: *SMART* (Bruker, 2001 ▶); cell refinement: *SAINT* (Bruker, 2001 ▶); data reduction: *SAINT*; program(s) used to solve structure: *SHELXS97* (Sheldrick, 2008 ▶); program(s) used to refine structure: *SHELXL97* (Sheldrick, 2008 ▶); molecular graphics: *SHELXTL* (Sheldrick, 2008 ▶); software used to prepare material for publication: *SHELXL97*.

## Supplementary Material

Crystal structure: contains datablock(s) I, global. DOI: 10.1107/S1600536812005752/kp2382sup1.cif

Structure factors: contains datablock(s) I. DOI: 10.1107/S1600536812005752/kp2382Isup2.hkl

Supplementary material file. DOI: 10.1107/S1600536812005752/kp2382Isup3.cml

Additional supplementary materials:  crystallographic information; 3D view; checkCIF report

## Figures and Tables

**Table 1:** Hydrogen-bond geometry (Å, °)

*D*—H⋯*A*	*D*—H	H⋯*A*	*D*⋯*A*	*D*—H⋯*A*
O3—H3⋯O1^i^	0.82	1.77	2.566 (2)	166
N2—H2*A*⋯O2	1.01 (2)	1.71 (2)	2.599 (2)	146.0 (16)

Symmetry code: (i) 
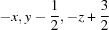
.

**Fig. 1.**
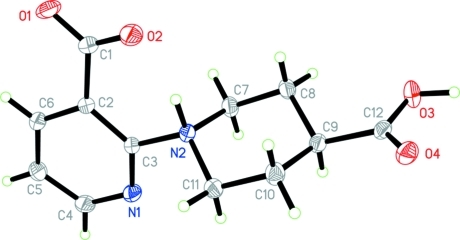
Molecular structure of the title compound, with displacement ellipsoids drawn at the 30% probability level(arbitrary sphere for the H atoms).

**Fig. 2.**
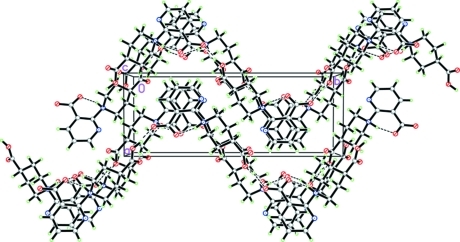
Packing diagram illustrating intra- and intermolecular hydrogen bonding interactions.

## supplementary crystallographic information

### Comment

The title compound, 2-(4-carboxylpiperdinium-1-yl)pyridine-3-carboxylic acid is known as one of 2-chloronicotinic acid derivatives (Fig. 1). It has attracted a great deal of interest in recent years. A series of inhibitors of human neutrophil elastase have been synthesised based on this derivative. The piperidine ring is in a chair conformation. By the intramolecular N2—H2A···O2 hydrogen bond, the dihedral angle between the ring defined by N1/C2—C6 and the plane defined by N2/C8/C10 of piperidine is 70.3 °. The dihedral angle between the ring defined by N1/C2—C6 and the ring defined by C7—C9—C11 of piperidine is 71.6 °. Molecules are linked by intermolecular O—H···O hydrogen bonds to form a chain (Table 1, Fig. 2). There are weak π-π interactions (Fig. 2) with the plane to plane distances of 3.69 Å and 3.72 Å (x,3/2-y,-1/2+z; 3,372-y, 1/2+z] which result in the formation of supramolecular network.

### Experimental

A mixture of 2-(4-methoxycarbonylpiperidin-1-yl)-3-pyridine formic acid ethyl ester (3.02 g, 10.7 mmol), NaOH (3.00 g, 75.0 mmol) and H_2_O (50 mL) was heated at reflux for 4 h. After cooling, the pH of resulting mixture was adjusted to 4–5 with dilute hydrochloric acid. After 12 h, a lot of white solid was precipitated. The precipitate was filtrated and dried, yield 73.9%, mp 481.8-483.2 K. Single crystal of the title compound was obtained by evaporating a solution of above-mentioned solid (0.2 mmol) in 11 mL ethanol.

### Refinement

H atoms attached to C atoms were positioned geometrically and refined using a riding model, with C*sp*^3^—H = 0.97 Å or C*sp*^2^—H = 0.93 Å and with *U*_iso_(H) = 1.2*U*_eq_(C). H atom attached to O atom was O—H = 0.82 Å and with *U*_iso_(H) = 1.5*U*_eq_(O). H atoms attached to N atom were located by difference Fourier synthesis and refined isotropically.

### Crystal data

**Table:**

C_12_H_14_N_2_O_4_	*F*(000) = 528
*M**_r_* = 250.25	*D*_x_ = 1.449 Mg m^−^^3^
Monoclinic, *P*2_1_/*c*	Mo *K*α radiation, λ = 0.71073 Å
Hall symbol: -P2ybc	Cell parameters from 361 reflections
*a* = 7.1094 (14) Å	θ = 2.5–22.7°
*b* = 18.667 (4) Å	µ = 0.11 mm^−^^1^
*c* = 8.6603 (17) Å	*T* = 293 K
β = 93.57 (3)°	Block, colourless
*V* = 1147.1 (4) Å^3^	0.20 × 0.20 × 0.20 mm
*Z* = 4	

### Data collection

**Table:**

Bruker SMART CCD area-detector diffractometer	2237 independent reflections
Radiation source: fine-focus sealed tube	1841 reflections with *I* > 2σ(*I*)
Graphite monochromator	*R*_int_ = 0.022
φ and ω scans	θ_max_ = 26.0°, θ_min_ = 2.2°
Absorption correction: multi-scan (*SADABS*; Bruker, 2001)	*h* = −8→8
*T*_min_ = 0.964, *T*_max_ = 0.983	*k* = −23→21
6010 measured reflections	*l* = −10→7

### Refinement

**Table:**

Refinement on *F*^2^	Primary atom site location: structure-invariant direct methods
Least-squares matrix: full	Secondary atom site location: difference Fourier map
*R*[*F*^2^ > 2σ(*F*^2^)] = 0.042	Hydrogen site location: inferred from neighbouring sites
*wR*(*F*^2^) = 0.118	H atoms treated by a mixture of independent and constrained refinement
*S* = 1.09	*w* = 1/[σ^2^(*F*_o_^2^) + (0.0685*P*)^2^ + 0.1385*P*] where *P* = (*F*_o_^2^ + 2*F*_c_^2^)/3
2237 reflections	(Δ/σ)_max_ = 0.001
168 parameters	Δρ_max_ = 0.22 e Å^−^^3^
0 restraints	Δρ_min_ = −0.34 e Å^−^^3^

### Special details

**Table:**

Geometry. All e.s.d.'s (except the e.s.d. in the dihedral angle between two l.s. planes) are estimated using the full covariance matrix. The cell e.s.d.'s are taken into account individually in the estimation of e.s.d.'s in distances, angles and torsion angles; correlations between e.s.d.'s in cell parameters are only used when they are defined by crystal symmetry. An approximate (isotropic) treatment of cell e.s.d.'s is used for estimating e.s.d.'s involving l.s. planes.
Refinement. Refinement of *F*^2^ against ALL reflections. The weighted *R*-factor *wR* and goodness of fit *S* are based on *F*^2^, conventional *R*-factors *R* are based on *F*, with *F* set to zero for negative *F*^2^. The threshold expression of *F*^2^ > σ(*F*^2^) is used only for calculating *R*-factors(gt) *etc*. and is not relevant to the choice of reflections for refinement. *R*-factors based on *F*^2^ are statistically about twice as large as those based on *F*, and *R*- factors based on ALL data will be even larger.

### Fractional atomic coordinates and isotropic or equivalent isotropic displacement parameters (Å^2^)

**Table:**

	*x*	*y*	*z*	*U*_iso_*/*U*_eq_	
O4	0.06398 (16)	0.42630 (6)	0.56128 (13)	0.0431 (3)	
N2	0.41209 (16)	0.63507 (6)	0.81715 (14)	0.0291 (3)	
O3	−0.10491 (16)	0.44848 (6)	0.76521 (13)	0.0445 (3)	
H3	−0.1719	0.4164	0.7277	0.067*	
N1	0.68999 (17)	0.64666 (6)	0.98026 (15)	0.0373 (3)	
O2	0.26774 (16)	0.75408 (6)	0.70841 (13)	0.0466 (3)	
C3	0.55581 (18)	0.68088 (7)	0.89789 (16)	0.0278 (3)	
C8	0.1114 (2)	0.56996 (8)	0.83901 (18)	0.0355 (4)	
H8A	0.0488	0.6001	0.7604	0.043*	
H8B	0.0187	0.5552	0.9102	0.043*	
C12	0.0444 (2)	0.45637 (7)	0.68272 (17)	0.0314 (3)	
O1	0.36031 (17)	0.85729 (6)	0.82086 (15)	0.0534 (4)	
C7	0.2667 (2)	0.61218 (8)	0.92594 (17)	0.0357 (4)	
H7A	0.2134	0.6541	0.9730	0.043*	
H7B	0.3256	0.5828	1.0077	0.043*	
C9	0.19275 (19)	0.50397 (7)	0.76289 (16)	0.0301 (3)	
H9	0.2557	0.4754	0.8458	0.036*	
C10	0.3436 (2)	0.52666 (8)	0.65609 (17)	0.0376 (4)	
H10A	0.4004	0.4843	0.6136	0.045*	
H10B	0.2858	0.5541	0.5707	0.045*	
C2	0.53838 (18)	0.75479 (7)	0.88389 (16)	0.0288 (3)	
C11	0.4954 (2)	0.57139 (8)	0.74008 (19)	0.0369 (4)	
H11A	0.5636	0.5422	0.8174	0.044*	
H11B	0.5842	0.5877	0.6670	0.044*	
C1	0.3745 (2)	0.79174 (8)	0.79697 (18)	0.0352 (4)	
C5	0.8281 (2)	0.76099 (9)	1.03995 (19)	0.0411 (4)	
H5	0.9268	0.7873	1.0871	0.049*	
C6	0.6822 (2)	0.79468 (8)	0.95705 (18)	0.0358 (4)	
H6	0.6801	0.8444	0.9501	0.043*	
C4	0.8245 (2)	0.68742 (9)	1.05140 (19)	0.0402 (4)	
H4	0.9201	0.6649	1.1114	0.048*	
H2A	0.344 (3)	0.6686 (11)	0.742 (2)	0.063 (6)*	

### Atomic displacement parameters (Å^2^)

**Table:**

	*U*^11^	*U*^22^	*U*^33^	*U*^12^	*U*^13^	*U*^23^
O4	0.0449 (6)	0.0350 (6)	0.0484 (7)	0.0028 (5)	−0.0044 (5)	−0.0133 (5)
N2	0.0281 (6)	0.0229 (6)	0.0362 (7)	−0.0020 (5)	−0.0001 (5)	0.0017 (5)
O3	0.0428 (6)	0.0447 (7)	0.0460 (7)	−0.0203 (5)	0.0027 (5)	−0.0074 (5)
N1	0.0333 (7)	0.0290 (7)	0.0483 (8)	0.0023 (5)	−0.0067 (6)	0.0049 (5)
O2	0.0421 (7)	0.0400 (6)	0.0551 (7)	0.0019 (5)	−0.0171 (6)	0.0043 (5)
C3	0.0244 (7)	0.0251 (7)	0.0340 (7)	−0.0014 (5)	0.0014 (6)	0.0008 (5)
C8	0.0297 (7)	0.0333 (8)	0.0438 (9)	−0.0055 (6)	0.0053 (6)	−0.0074 (6)
C12	0.0356 (8)	0.0207 (7)	0.0370 (8)	0.0022 (6)	−0.0039 (6)	0.0021 (6)
O1	0.0497 (7)	0.0295 (6)	0.0787 (9)	0.0131 (5)	−0.0153 (6)	−0.0008 (5)
C7	0.0333 (8)	0.0342 (8)	0.0404 (8)	−0.0071 (6)	0.0090 (6)	−0.0082 (6)
C9	0.0318 (7)	0.0257 (7)	0.0323 (7)	−0.0019 (6)	−0.0016 (6)	0.0019 (6)
C10	0.0390 (8)	0.0356 (8)	0.0389 (8)	−0.0056 (6)	0.0078 (7)	−0.0078 (6)
C2	0.0264 (7)	0.0252 (7)	0.0350 (8)	0.0014 (5)	0.0036 (6)	0.0008 (5)
C11	0.0328 (7)	0.0331 (8)	0.0457 (9)	−0.0026 (6)	0.0089 (6)	−0.0053 (7)
C1	0.0325 (7)	0.0302 (8)	0.0426 (8)	0.0039 (6)	−0.0006 (6)	0.0039 (6)
C5	0.0309 (8)	0.0379 (9)	0.0532 (10)	−0.0054 (6)	−0.0082 (7)	−0.0037 (7)
C6	0.0327 (7)	0.0257 (7)	0.0488 (9)	−0.0022 (6)	0.0006 (7)	−0.0010 (6)
C4	0.0303 (7)	0.0396 (9)	0.0491 (9)	0.0048 (6)	−0.0105 (7)	0.0037 (7)

### Geometric parameters (Å, º)

**Table:**

O4—C12	1.2079 (18)	C7—H7A	0.9700
N2—C3	1.4749 (17)	C7—H7B	0.9700
N2—C11	1.5030 (18)	C9—C10	1.519 (2)
N2—C7	1.5031 (18)	C9—H9	0.9800
N2—H2A	1.01 (2)	C10—C11	1.515 (2)
O3—C12	1.3240 (18)	C10—H10A	0.9700
O3—H3	0.8200	C10—H10B	0.9700
N1—C3	1.3200 (18)	C2—C6	1.386 (2)
N1—C4	1.342 (2)	C2—C1	1.514 (2)
O2—C1	1.2593 (18)	C11—H11A	0.9700
C3—C2	1.3897 (19)	C11—H11B	0.9700
C8—C7	1.518 (2)	C5—C6	1.376 (2)
C8—C9	1.528 (2)	C5—C4	1.377 (2)
C8—H8A	0.9700	C5—H5	0.9300
C8—H8B	0.9700	C6—H6	0.9300
C12—C9	1.5142 (19)	C4—H4	0.9300
O1—C1	1.2462 (18)		
			
C3—N2—C11	112.86 (11)	C10—C9—H9	106.9
C3—N2—C7	110.53 (11)	C8—C9—H9	106.9
C11—N2—C7	111.03 (11)	C11—C10—C9	111.73 (12)
C3—N2—H2A	103.8 (11)	C11—C10—H10A	109.3
C11—N2—H2A	113.0 (11)	C9—C10—H10A	109.3
C7—N2—H2A	105.2 (11)	C11—C10—H10B	109.3
C12—O3—H3	109.5	C9—C10—H10B	109.3
C3—N1—C4	116.38 (13)	H10A—C10—H10B	107.9
N1—C3—C2	125.81 (13)	C6—C2—C3	115.73 (13)
N1—C3—N2	115.57 (12)	C6—C2—C1	120.38 (12)
C2—C3—N2	118.61 (12)	C3—C2—C1	123.88 (13)
C7—C8—C9	110.46 (12)	N2—C11—C10	111.12 (12)
C7—C8—H8A	109.6	N2—C11—H11A	109.4
C9—C8—H8A	109.6	C10—C11—H11A	109.4
C7—C8—H8B	109.6	N2—C11—H11B	109.4
C9—C8—H8B	109.6	C10—C11—H11B	109.4
H8A—C8—H8B	108.1	H11A—C11—H11B	108.0
O4—C12—O3	123.91 (14)	O1—C1—O2	126.68 (14)
O4—C12—C9	123.86 (13)	O1—C1—C2	115.64 (13)
O3—C12—C9	112.14 (12)	O2—C1—C2	117.68 (12)
N2—C7—C8	110.12 (12)	C6—C5—C4	118.46 (14)
N2—C7—H7A	109.6	C6—C5—H5	120.8
C8—C7—H7A	109.6	C4—C5—H5	120.8
N2—C7—H7B	109.6	C5—C6—C2	120.24 (14)
C8—C7—H7B	109.6	C5—C6—H6	119.9
H7A—C7—H7B	108.1	C2—C6—H6	119.9
C12—C9—C10	112.48 (11)	N1—C4—C5	123.25 (14)
C12—C9—C8	113.54 (12)	N1—C4—H4	118.4
C10—C9—C8	109.78 (11)	C5—C4—H4	118.4
C12—C9—H9	106.9		

### Hydrogen-bond geometry (Å, º)

**Table:**

*D*—H···*A*	*D*—H	H···*A*	*D*···*A*	*D*—H···*A*
O3—H3···O1^i^	0.82	1.77	2.566 (2)	166
N2—H2*A*···O2	1.01 (2)	1.71 (2)	2.599 (2)	146.0 (16)

Symmetry code: (i) −*x*, *y*−1/2, −*z*+3/2.

## References

[bb1] Bonnet, V., Mongin, F., Trécourt, F., Quéguiner, G. & Knochel, P. (2002). *Tetrahedron*, **58**, 4429–4438.

[bb2] Bruker (2001). *SMART*, *SAINT* and *SADABS* Bruker AXS Inc., Madison, Wisconsin, USA.

[bb3] Léost, F., Chantegrel, B. & Deshayes, C. (1997). *Tetrahedron*, **53**, 7557–7576.

[bb4] Sheldrick, G. M. (2008). *Acta Cryst.* A**64**, 112–122. 10.1107/S0108767307043930 18156677

[bb5] Shreder, K. R., Cajica, J., Du, L., Fraser, A., Hu, Y. & Kohno, Y. (2009). *Bioorg. Med. Chem. Lett.* **19**, 4743–4746. 10.1016/j.bmcl.2009.06.053 19577470

